# Case Report: Laparoscopic management of penetrating liver injury from pneumatic nail gun

**DOI:** 10.3389/fsurg.2025.1706137

**Published:** 2025-12-10

**Authors:** Mingyu Zhang, Xuchi Li, Qingbo Feng

**Affiliations:** 1 Department of General Surgery, Dejiang County People’s Hospital, Tongren, China; 2 Department of General Surgery, Digestive Disease Hospital, Affiliated Hospital of Zunyi Medical University, Zunyi, Guizhou, China

**Keywords:** pneumatic nail gun, liver, laparoscopic surgery, case report, liver injury

## Abstract

Foreign bodies in the liver are relatively rare, with most cases resulting from ingested objects that migrate through the gastrointestinal tract. Direct hepatic penetration by pneumatic tools represents an exceptionally rare mechanism of injury, as these foreign bodies typically require significant force to breach the abdominal wall and lodge within the liver parenchyma. Preoperative differentiation between migrated and directly penetrating foreign bodies is clinically significant, as it may influence both diagnostic approach and surgical planning. Here, we report the diagnosis and management of a 50-year-old male with an unusual case of pneumatic nail penetration into the left hepatic lobe. The patient presented with acute abdominal pain following accidental penetration by a 5.2 cm pneumatic nail that traversed the rectus abdominis to lodge in segment III of the liver. CT imaging confirmed the nail's trajectory while demonstrating no evidence of abscess formation or pneumoperitoneum. Vital signs and laboratory parameters remained stable throughout the clinical course. During exploratory laparoscopy, the foreign body was identified within the left hepatic lobe, confirming its direct penetration pathway rather than migratory origin. The nail was successfully extracted using careful laparoscopic dissection, with complete preservation of surrounding parenchyma and no significant bleeding. The patient experienced an uneventful recovery and was discharged on postoperative day 2 with complete resolution of symptoms.

## Introduction

For gastrointestinal foreign bodies, the majority of ingested foreign bodies can pass through the digestive tract spontaneously without the need for intervention (1). However, some of the foreign bodies are sharp and rigid, which may penetrate the walls of the stomach or duodenum and migrate to surrounding organs such as the pancreas and liver (2). These foreign bodies usually include chicken bones, fish bones, sewing needles and so on, which may cause abscess, pseudoaneurysm, pancreatitis, and high-mortality-risk complications (1–3). When conditions are permitted, laparoscopic surgery can be used to remove the foreign bodies (1). Here we report the case of a rare case of liver injury caused by a pneumatic nail gun that penetrated the abdominal wall and embedded in the liver, successfully managed with laparoscopic removal.

## Case presentation

A50-year-old male construction worker who presented to our emergency department following an occupational accident involving a pneumatic nail gun. The patient, with no significant medical history, sustained a penetrating abdominal injury approximately 90 min prior to admission when a nail was accidentally discharged into his abdomen during routine construction work.

Upon arrival, the patient was hemodynamically stable with normal vital signs: temperature 36.8°C, pulse 78 beats/min, blood pressure 134/87 mmHg, respiratory rate 18 breaths/min, and oxygen saturation of 98% on room air. Physical examination revealed a 0.2 cm circular entry wound in the right upper quadrant. The abdomen was mildly tender locally but without signs of peritoneal irritation, and bowel sounds were normal in all quadrants.

**Figure 1:**
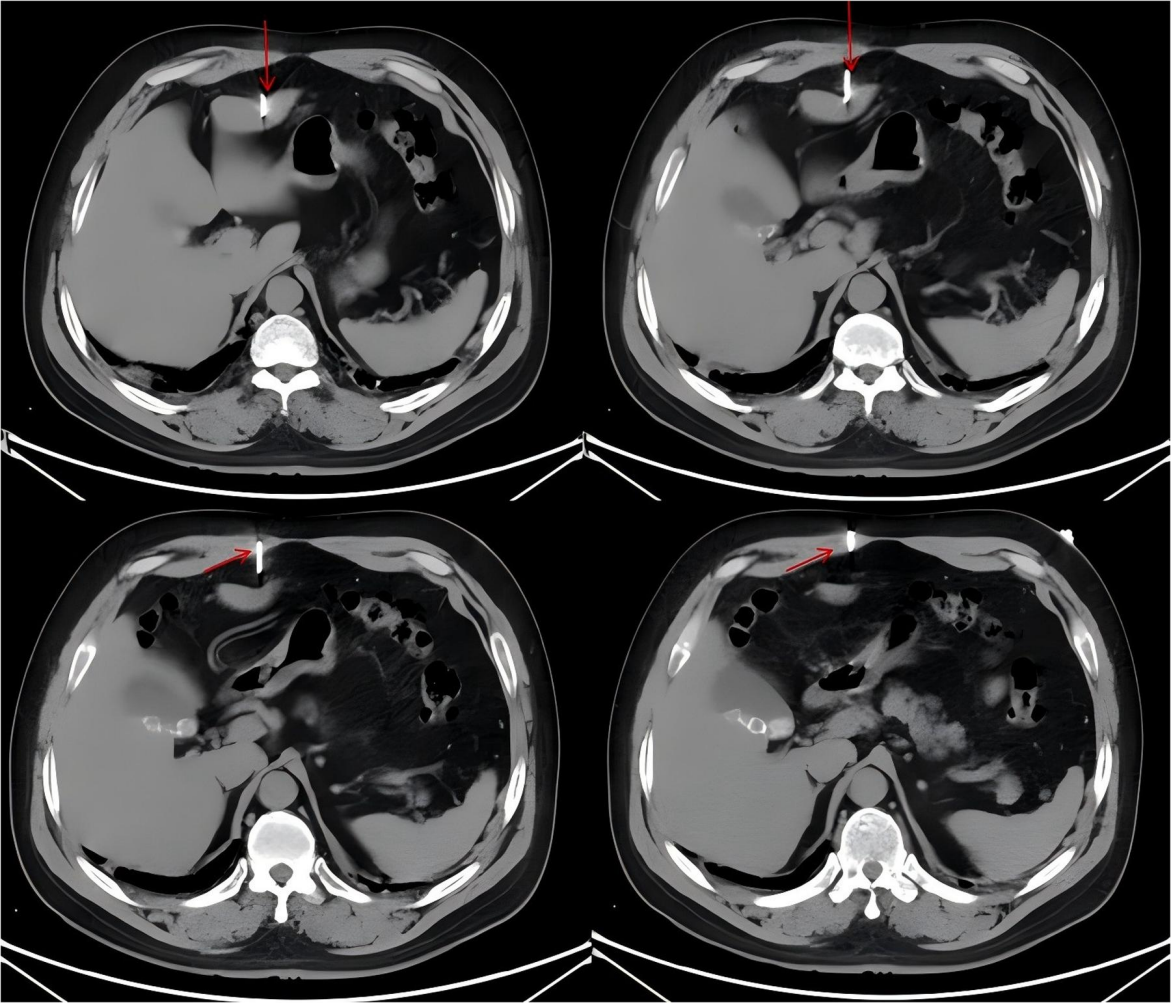
Abdominal CT scan demonstrating penetrating nail injury, red arrow shows the gun nail.

Laboratory investigations showed entirely normal results, including complete blood count, liver function tests, and coagulation profile. Abdominal CT scan demonstrated a 5.2 cm linear metallic foreign body (nail) traversing from the right rectus abdominis muscle through to the left hepatic lobe (Figure 1). The imaging revealed minimal perilesional edema in the liver parenchyma without evidence of pneumoperitoneum, active bleeding, significant hemoperitoneum, or biliary tree injury. Adjacent organs including the stomach, pancreas, and kidneys appeared normal.

Following multidisciplinary consultation, the patient underwent emergency laparoscopic exploration under general anesthesia. The surgical procedure began with establishing pneumoperitoneum at 12 mmHg via Veress needle, followed by placement of three trocars: a 10 mm umbilical port for the camera and two 5 mm working ports in the right and left midclavicular lines. Systematic abdominal exploration confirmed the nail's trajectory through the rectus abdominis with its tip embedded approximately 1 cm into liver segment III. The operative field contained minimal hemoperitoneum with no other visceral injuries identified.The Pringle manoeuvre was not employed in this case due to the specific nature and location of the injury, which did not necessitate hepatic inflow occlusion. Low-central venous pressure (CVP) anaesthesia was also not used, as the patient's hemodynamic status was stable throughout the procedure, and there was no indication for its use. A surgical drain was placed intraoperatively to monitor for any potential bleeding or bile leakage. The drain output was minimal and consisted primarily of serous fluid, with no evidence of bile or blood. The drain was removed on postoperative day 3, as there was no significant output and the patient was clinically stable.

**Figure 2:**
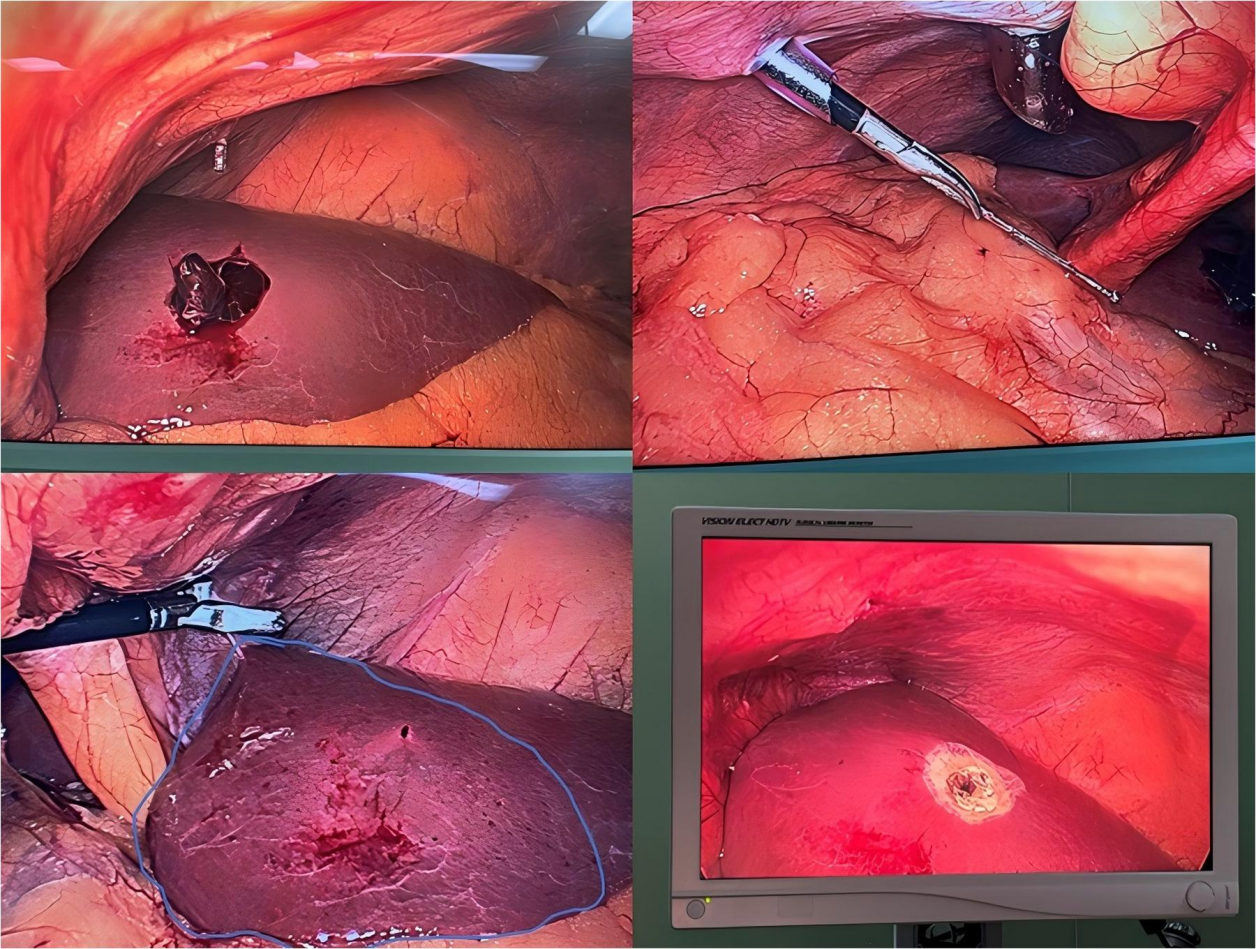
Intraoperative images during the laparoscopic exploration and nail extraction procedure.

The extraction procedure involved meticulous dissection of surrounding adhesions followed by gradual withdrawal of the nail under direct visualization.The extracted nail measured 5.2 cm in length and 3 mm in diameter, with a smooth surface devoid of barbs and minimal tissue adherence. The liver injury was classified as Grade I parenchymal laceration without active bleeding or bile staining (Figure 2).

As shown in Table 1, postoperative recovery was entirely uneventful. The patient was monitored in the surgical ward with NPO orders and IV fluids on postoperative day 0, progressing to clear liquids on day 1 when he ambulated independently. On the second day after the surgery, a liver function test was conducted for re—examination. The results showed ALT 15 U/L,AST 20 U/L,total bilirubin 7.5 umol/L,GGT 7 U/L. He tolerated a regular diet, allowing for discharge with oral analgesics. Follow-up at 1 week showed excellent wound healing.Three months later, through telephone follow—up, the patient reported that everything was fine and they had returned to normal work.

**Table 1:** A timeline table covering: time of injury, ED arrival, CT acquisition, operation, POD 0-2 milestones, 1-week and 3-month outcomes.

Time/event	Details
Time of Injury	[July 15th, 2024 14:20] Sustained blunt abdominal trauma, potentially causing internal organ damage.
ED Arrival	[July 15th, 2024 14:50] Presented with severe abdominal pain, nausea, and vomiting. Primary survey initiated.
CT Acquisition	[July 15th, 2024 15:30] Abdominal CT showed an air nail has penetrated the liver.
Operation	[Start: July 15th, 2024 19:00; End: July 15th, 2024 19:30] Laparoscopic exploration. Successfully removed the air nail from the abdominal cavity.
POD 0	Returned to the general ward after surgery. Continuous monitoring of vital signs was carried out.
POD 1	The patient passed gas. After that, clear liquid intake was initiated as tolerated.
POD 2	The patient’s condition has recovered well. Subsequently, discharge was approved.
1 - Week Outcome	Incision healing well.
3 - Month Outcome	No discomfort. Resumed normal activities and work. No long - term complications.

## Discussion

The management of penetrating abdominal injuries caused by foreign bodies presents unique clinical challenges that require careful consideration of multiple factors. While the majority of accidentally ingested foreign bodies pass spontaneously through the gastrointestinal tract, a small but significant proportion can penetrate the gut wall and migrate to adjacent organs, as evidenced by numerous case reports in the medical literature (4, 5). This phenomenon is particularly concerning given the often subtle and nonspecific nature of symptoms, which frequently mimic common conditions like gastroesophageal reflux or gastritis, leading to potential delays in diagnosis and treatment.

The current case of a pneumatic nail gun injury to the liver represents a distinct clinical scenario that differs from typical foreign body ingestion cases in several important aspects. Unlike swallowed objects that migrate through the digestive system, industrial tool injuries involve direct mechanical penetration with different biomechanical properties and injury patterns. Pneumatic nail guns can propel fasteners at velocities exceeding 100 m/s, generating sufficient kinetic energy to penetrate multiple tissue layers while often creating a relatively clean wound channel due to the smooth surface of industrial nails.

Computed tomography (CT) imaging has emerged as the diagnostic modality of choice for such cases, offering several critical advantages over conventional radiography. The ability to clearly visualize linear hyperdense foreign bodies in three dimensions provides invaluable information about their precise location, trajectory, and relationship to vital structures (6, 7). In the present case, CT not only confirmed the presence and position of the nail but also ruled out complications such as pneumoperitoneum or significant hemoperitoneum, which significantly influenced the treatment approach. The modality's capacity to assess soft tissue injury and detect subtle signs of vascular or biliary involvement makes it indispensable for surgical planning and prognostication.

The therapeutic approach to penetrating foreign bodies has evolved considerably with advances in minimally invasive techniques. While endoscopic removal remains the preferred method for many gastrointestinal foreign bodies, its utility is limited in cases of complete transmural penetration or extra-luminal migration. Laparoscopic surgery has demonstrated particular advantages in selected cases, offering the benefits of minimally invasive access while maintaining the capability for thorough abdominal exploration and definitive treatment (8–10). The current case illustrates these advantages well, with successful extraction of the nail, assessment of associated injuries, and repair of tissue defects all accomplished through small incisions.

Several technical considerations proved crucial in the laparoscopic management of this case. The extraction technique required careful attention to the nail's path to avoid iatrogenic injury to surrounding structures. The smooth surface of the industrial nail facilitated removal without tissue drag or fragmentation, contrasting with the challenges posed by barbed or irregular foreign bodies. Intraoperative assessment of hemostasis and biliary integrity was particularly important given the liver involvement, and the use of topical hemostatic agents complemented precise bipolar cautery to achieve secure hemostasis.

From a technical standpoint, the case underscores the importance of proper port placement and instrument selection in laparoscopic foreign body removal. The use of angled laparoscopes and atraumatic graspers facilitated safe manipulation of the nail while minimizing tissue trauma. The decision to place a drain, while not universally necessary in such cases, provided an additional safety measure for monitoring potential bleeding or bile leakage in the early postoperative period.

The successful outcome in this case was facilitated by several favorable factors, including the patient's hemodynamic stability, the contained nature of the injury, and the absence of hollow viscus involvement. These characteristics made the patient an ideal candidate for minimally invasive management, though it's important to recognize that not all penetrating foreign body injuries will be suitable for this approach. Cases with hemodynamic instability, significant contamination, or complex multivisceral involvement may still require traditional open exploration.

### Limitations

Our study, while providing valuable insights into the specific case at hand, is not without its limitations. First and foremost, it is a single—case report, lacking a control group for comparison. This inherent characteristic significantly restricts the generalisability of our findings. The unique circumstances and individual factors associated with this single patient may not be representative of a broader population, making it challenging to apply our results to other similar cases or draw widespread conclusions. Moreover, the follow—up period in our study was limited to only 3 months. This relatively short time frame may not be adequate to identify all potential long—term complications. Some complications, especially those that develop gradually over an extended period, might have gone undetected during this 3 - month follow—up. As a result, our understanding of the long—term prognosis and safety of the treatment or intervention described in our study remains incomplete.

In conclusion, this case of laparoscopic management of a pneumatic nail gun injury to the liver demonstrates the potential benefits of minimally invasive techniques in selected cases of penetrating abdominal trauma. The approach allowed for definitive treatment with minimal morbidity, rapid recovery, and excellent functional outcomes. The case highlights the importance of careful patient selection, precise preoperative imaging, and meticulous surgical technique in achieving optimal results. As minimally invasive techniques continue to evolve, they are likely to play an increasingly prominent role in the management of abdominal foreign body injuries across a spectrum of clinical scenarios.

## Data Availability

The original contributions presented in the study are included in the article/Supplementary Material, further inquiries can be directed to the corresponding author.
